# Stresses and Strains in Cruciform Samples Deformed in Tension

**DOI:** 10.1007/s11340-017-0270-6

**Published:** 2017-04-27

**Authors:** M. V. Upadhyay, T. Panzner, S. Van Petegem, H. Van Swygenhoven

**Affiliations:** 1 Swiss Light Source, Paul Scherrer Institute, CH-5232 Villigen PSI, Switzerland; 2 Laboratory for neutron scattering, NUM, Paul Scherrer Institute, CH-5232 Villigen PSI, Switzerland; 3 Neutrons and X-rays for Mechanics of Materials, IMX, Ecole Polytechnique Fédérale de Lausanne, CH-1012 Lausanne, Switzerland

**Keywords:** Cruciform, Biaxial stress, Finite elements, Plastic strain, Stress concentration

## Abstract

The stress and strain relationship in the gauge region of six cruciform geometries is studied: the ISO standard geometry with slits in arms, two geometries with thinned gauge areas, two geometries with thinned gauge areas and slits in arms, and one modified ISO standard geometry with slits in arms and a thinned gauge area. For all the geometries, finite element simulations are performed under uniaxial loading to compare the plastic strain, the von Mises stress distribution and the in-plane stress evolution. Results show that less plastic strain can be achieved in the gauge of the two ISO standard geometries. For the remaining cruciform geometries, a strong non-linear coupling between applied forces in arms and gauge stresses is generated. The evolution of this non-linear coupling depends on the geometry type, applied biaxial load ratio and the elastic-plastic properties of the material. Geometry selection criteria are proposed to reduce this non-linear coupling.

## Introduction

Sheet metals and alloys are subjected to biaxial stresses and changing strain paths during their forming processes and under service conditions. Under these conditions, the mechanical properties can differ from those obtained under uniaxial stress conditions. Several biaxial deformation devices have been developed to test materials, each of them having advantages and disadvantages [1, 2]. Amongst these, biaxial deformation rigs using cruciform shaped samples allow to deform materials under different in-plane loading modes [2–5]. The cruciform shape offers the possibility for applying any arbitrary load ratio, thus providing access to a large portion of the 2-dimensional stress space. It allows full-field in-situ strain measurements on either side of the gauge region, in contrast to Marciniak samples, punch test samples, and tubular samples, whose internal sides are inaccessible for techniques such as digital image correlation (DIC). Additionally, when the axes of the device can operate independently, it allows to perform non-proportional strain path changes [6–8]. It should be noted that the eigenstress coordinate system cannot be changed during non-proportional loadings, thus making it difficult to perform in-depth analysis of non-proportional strain path change tests. Furthermore, when biaxial testing is performed in-situ during neutron or X-ray diffraction, footprints of the microstructural evolution such as texture, inter and intra-granular stresses and dislocation density can be followed [9–11]. The cruciform geometry also has its drawbacks. One of the major challenges is to develop a cruciform geometry that can achieve significant amount of plastic deformation in the gauge section while allowing analytical computation of the gauge stresses.

**Fig. 1:**
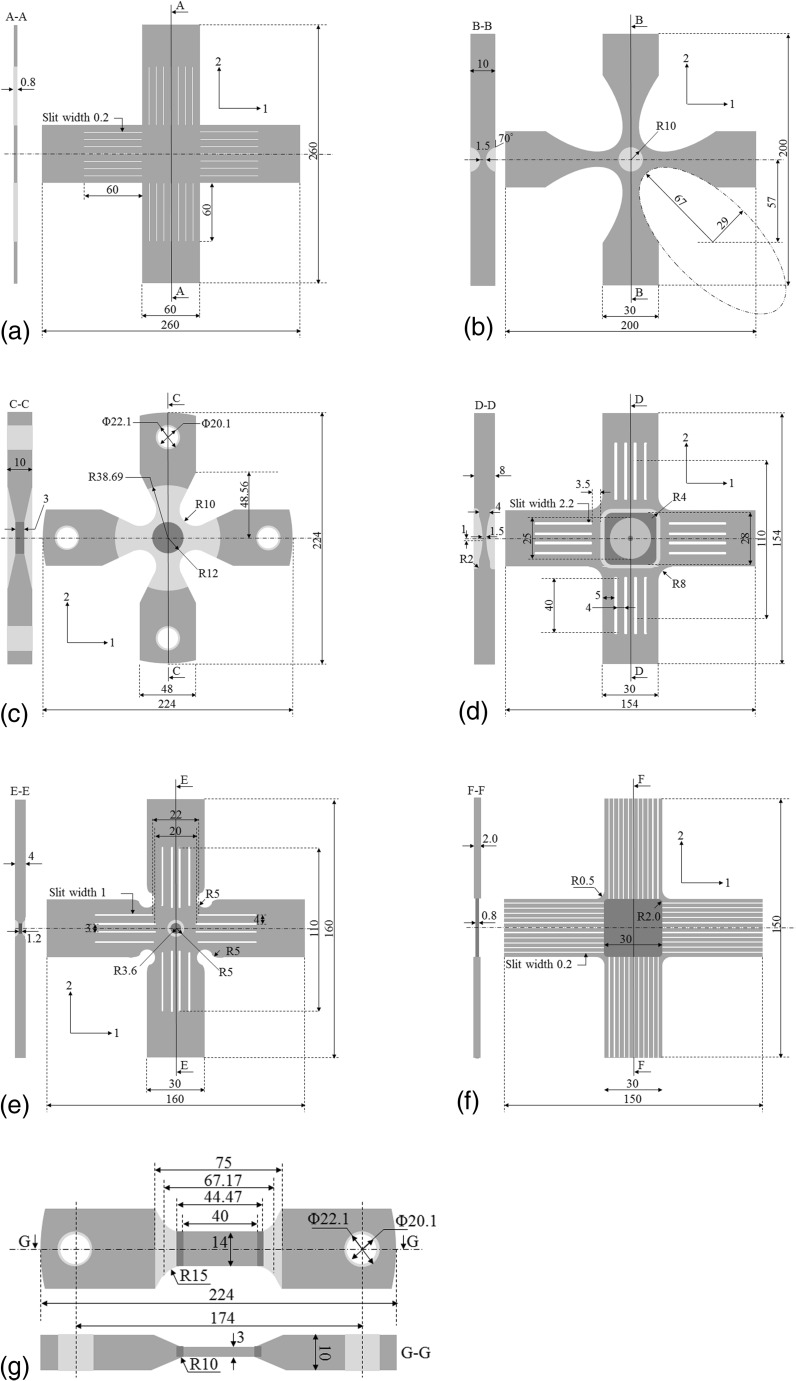
Cruciform geometries: (a) ISO standard slit geometry [2, 17, 18] – SLIT-I, (b) the elliptical cross-arm steeply thinned geometry with no slits [24] – THIN-I, (c) the circular cross-arm gradually thinned geometry with no slits [10, 28, 29] – THIN-II, (d) the two-step gradually thinned geometry with slits [8, 30] – SLIT-THIN-I, (e) the uneven slit, circular notched and sharply thinned geometry [31] – SLIT-THIN-II, (f) the modified ISO standard slit geometry [32] – SLIT-THIN-III, and (g) the dog-bone geometry [10] – DB

To that end, significant efforts have been directed towards optimizing the cruciform geometry. Recently, an ISO standard [12] for biaxial tensile testing was established. According to this standard, the cruciform design should have a uniform thickness and slits in the arms, as shown in Fig. 1(a). The slit based design was originally proposed in the work of Hayhurst [13], and later adopted by Kelly [14], Makinde et al. [15, 16], and Kuwabara and co-workers [2, 17, 18]. The purpose of the slits is two-fold: (i) to reduce the stress heterogeneity within the square gauge area so that the in-plane normal stress components can be computed as the force divided by area along a given direction, and (ii) to prevent shear loadings in the cruciform arms or machine grips in case the machine is not perfectly aligned. This geometry has been used in several studies [19–21] to perform non-proportional strain path changes and determine yield surface evolution. One of the main drawbacks of this design is that large stress concentrations can develop at the slit-ends during uniaxial or biaxial loading. Consequently, the amount of plastic strain achieved in the gauge region is low. Another drawback was revealed in the FE simulations performed by Hanabusa et al. [22], who showed the presence of in-plane compressive stresses normal to the uniaxial loading direction. Their influence on the computation of stresses as force divided by area was not addressed. Not accounting for this may affect the prediction of yield surface and its evolution for different material systems.

It is well known that the achievable amount of gauge plastic strain can be increased by locally thinning the gauge section [3, 23]. Two such thinned geometries are presented in the Figs. 1(b) and (c). The cruciform geometry in Fig. 1(b) was proposed in the work of Baptista et al. [24]. They used an optimization procedure to obtain (i) the shape of the intersection of cruciform arms (henceforth known as cross-arms) and (ii) the depth of thinned region, for a given thickness of the sheet material. The gauge area was continuously thinned down using a spline curve. In contrast, the geometry in Fig. 1(c) has a constant thickness in the gauge region and a gradual thickness reduction from the arms to the gauge region. Thinning of the cruciform sample, however, does not facilitate fulfilling the other requirements, i.e. a homogeneous stress distribution and a correct analytical computation of the gauge stresses. Makinde and co-workers [15, 16, 25] were the first to argue that computing gauge stresses as force divided by area may be erroneous for some cruciform geometries because of the difficulty in accurately determining the cross-sectional gauge areas. Based on this argument, Green et al. [26] suggested the use of finite element (FE) simulations to obtain the gauge stresses. They proposed an iterative procedure to match the simulation predicted forces in the arms and the gauge strains with experimental data. Simulation predicted gauge stress vs strain plots were obtained for different yield functions. The evolution of gauge stresses as a function of the forces in the arms was however not reported. Hoferlin et al. [27] demonstrated, for their cruciform geometry, that a uniaxial load in one of the arms results in a biaxial stress state in the gauge region. Furthermore, when loading with in-plane force ratios between 0 and 0.36, the in-plane stress component corresponding to the lower load is significantly under predicted when computed as force divided by area. For a geometry similar to the one in Fig. 1(c), Bonnand et al. [28] proposed to relate the applied forces (*F*_1_ and *F*_2_) in the arms and gauge stresses (*S*_11_ and *S*_22_) using a linear relationship: *S*_11_ = *aF*_1_ − *bF*_2_ and *S*_22_ =  − *bF*_1_ + *aF*_2_, with *a* and *b* as constants. A similar relationship was already proposed in [27]. Claudio et al. [29] estimated the values of *a* and *b* from FE simulations of elastic deformation under uniaxial tension and used them to compute the stresses for all load ratios. Such a linear coupling between forces and stresses is however only valid in the elastic regime [27]. For plastically deforming materials, this coupling will be inherently non-linear.

Cruciform geometries combining slits and thinner gauge areas have also been proposed. One such geometry was introduced in the work of Zidane et al. [30], similar to the one shown in Fig. 1(d). This geometry has four equal-length slits per arm. It has a width that is twice the gauge thickness, and is more than an order of magnitude higher than the ISO standard geometry. Similar to the ISO standard geometry, there are no notches in the arms but the cross-arm radius of curvature is much higher. A two-step thickness reduction is introduced on one side of the cruciform in the gauge region; first a square zone with an abrupt thickness reduction is introduced, then a second circular zone is added with a gradual thickness reduction. This specimen was used to obtain forming limit curves for aluminum alloy AA5086. Fracture always occurred at the center of the sample independent of the loading conditions. Leotoing et al. [8] used this geometry to developed a predictive numerical model for the forming limit curve using FE simulations. In contrast, Liu et al. [31] introduced slits with different lengths in their cruciform geometry, circular notches at cross-arms with smooth ends at the arms and a steep transition to the gauge area (Fig. 1(e)). Deng et al. [32] proposed a modified slit design (Fig. 1(f)), that conforms to the ISO standard specifications, in order to increase the amount of plastic deformation prior to failure while keeping the gauge shape for the analytical computation of the stresses. Similar to the works of Hayhurst [13] and Kelly [14], the arm thickness is increased with respect to the gauge region. Equi-spaced slits are drawn over the entire length of the arms. A comparison of the plastic strain reached in the gauge area and at the location of stress concentrations is not reported. Furthermore, the role of cruciform geometry on the coupling between force in the arms and gauge stresses has not been discussed for any of the slit-thinned geometries.

Understanding the non-linear coupling between forces and stresses is important since they can significantly affect the inter- (type II) and intra- (type III) granular stress state and microstructural evolution. In a recent work involving the authors [10], in-situ neutron diffraction studies during biaxial strain path changes were performed on 316 L stainless steel cruciform samples having the geometry shown in Fig. 1(c). During monotonic uniaxial tensile loading, the presence of significant in-plane compressive stresses normal to the loading direction was confirmed using DIC strain measurements and FE simulations. Furthermore, in the plastic regime the evolution of diffraction peak positions i.e. microscopic strains associated with type I stresses (applied stresses), for different grain families as a function of the stress was significantly different from that obtained for dog-bone (DB) samples. Similar cruciform geometric effects in DIC strains were also observed by [9] during in-situ x-ray diffraction studies of biaxial deformation of their ferritic sheet steel cruciform samples (not shown here).

In light of the above, the main objective of the present work is to highlight the role of cruciform geometry on the gauge stress evolution during elastic and plastic deformation under monotonic uniaxial and biaxial tensile loading. FE simulations are performed on six cruciform geometries shown in Fig. 1, i.e. (i) the ISO standard slit geometry [2, 17, 18], (ii) the elliptical cross-arm steeply thinned geometry without slits [24], (iii) the circular cross-arm gradually thinned geometry without slits [10, 28, 29], (iv) the two-step gradually thinned geometry with slits [8], (v) the uneven slit, circular notched and sharply thinned geometry [31], and (vi) the modified ISO standard slit geometry [32]. These are classified according to the presence of slits and/or gauge thinning as SLIT-I, THIN-I, THIN-II, SLIT-THIN-I, SLIT-THIN-II, SLIT-THIN-III, respectively. The results are compared with those obtained for DB samples used in [10]. The remainder of this paper is divided as follows. Section 2 describes the experimental procedure and section 3 the FE simulation setup. Section 4 begins with the experimental validation of the simulation framework using the THIN-II geometry. Then the equivalent plastic strain and von Mises stress distributions in the six cruciform geometries are plotted to study the stress concentrations and maximum achievable plastic strains for each geometry. Next, the in-plane gauge stress evolution for the six cruciform geometries is analyzed and compared with the results of DB samples. The THIN-II geometry is then studied for different biaxial load ratios and different materials. The results of these simulations are used to propose geometry selection criteria in section 5. The main conclusions of this study are presented in section 6.

## Experimental Procedure

THIN-II and DB samples are deformed using a biaxial deformation rig developed in collaboration with Zwick/Roell (Ulm, Germany) [6, 10]. A detailed description of the machine is presented in [10]; for brevity, only the relevant details of the machine are recalled here. The biaxial testing rig is equipped with independent arm control and allows to deform up to 50 kN along direction 1 and 100kN along direction 2. A two-camera ARAMIS4M DIC system from GOM is installed on the biaxial rig to measure the surface strains on the gauge region of the samples. The error associated with the DIC measurement for the THIN-II and DB samples is given using the equation *err*(%) = *x* x strain(%) + *y*; where *x* and *y* are in the range [0.014, 0.024] and [0.05, 0.09], respectively.

**Fig. 2:**
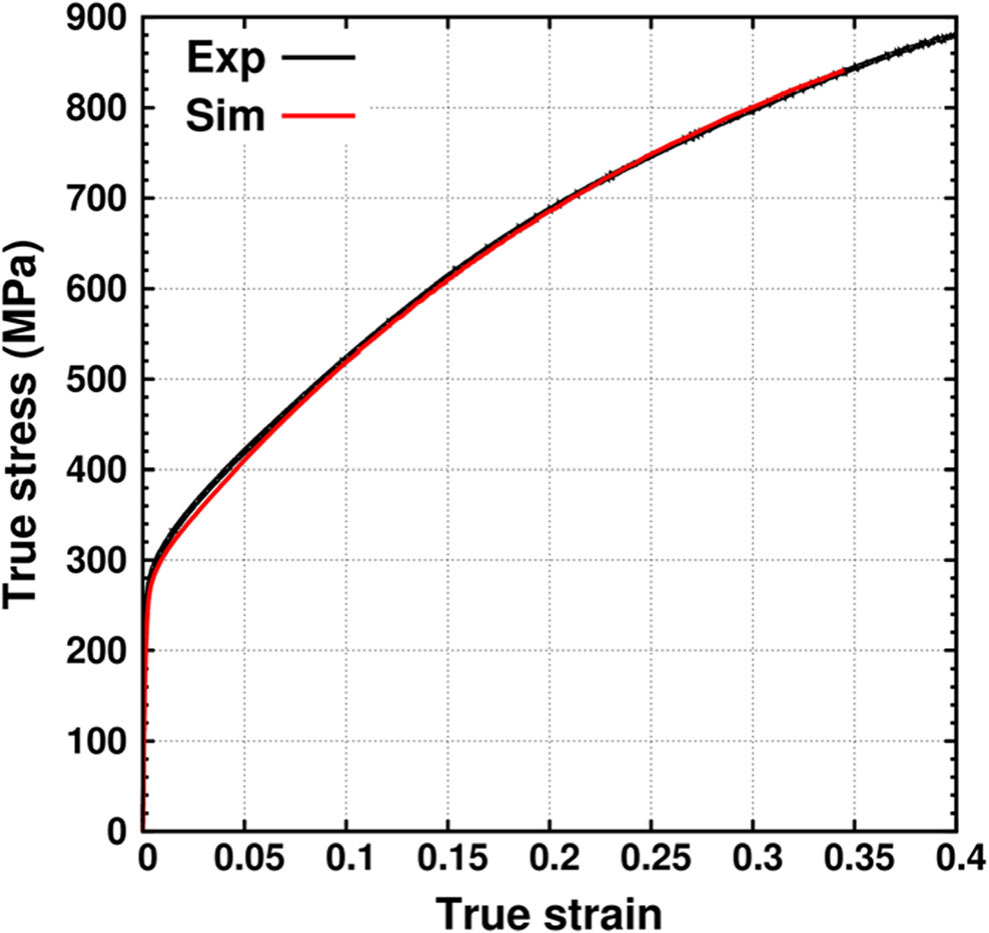
True stress v/s true strain curve for uniaxial tensile loading of DB samples from experiments (Exp) and simulations (Sim)

The test material is a warm rolled 10 mm thick sheet of 316 L stainless steel with composition (in %wt) 17.25Cr, 12.81Ni, 2.73Mo, 0.86Mn, 0.53Si and 0.02C. An electron backscattering diffraction analysis of this steel reveals a mild texture, but the uniaxial mechanical response in rolling and transverse direction were the same as was reported in [10]. This implies that the macroscopic uniaxial mechanical response of the material can be assumed to be independent of the rolling direction. The isotropic elastic Young’s modulus (Y) is 190 GPa and the Poisson’s ratio (*ν*) is 0.31. Figure 2 shows the true stress v/s true strain curve (in black) for this steel obtained from uniaxial tests on DB samples (different from the one shown in Fig. 1(g)) performed by Dr. Niffenegger (see acknowledgements).

The THIN-II and DB samples are prepared from this steel. The outer geometry of the THIN-II and DB samples is water cut and the gauge section is mechanically ground in a symmetric manner to a thickness of 3 mm. Mechanical tests have not been performed for the remaining geometries.

**Fig. 3:**
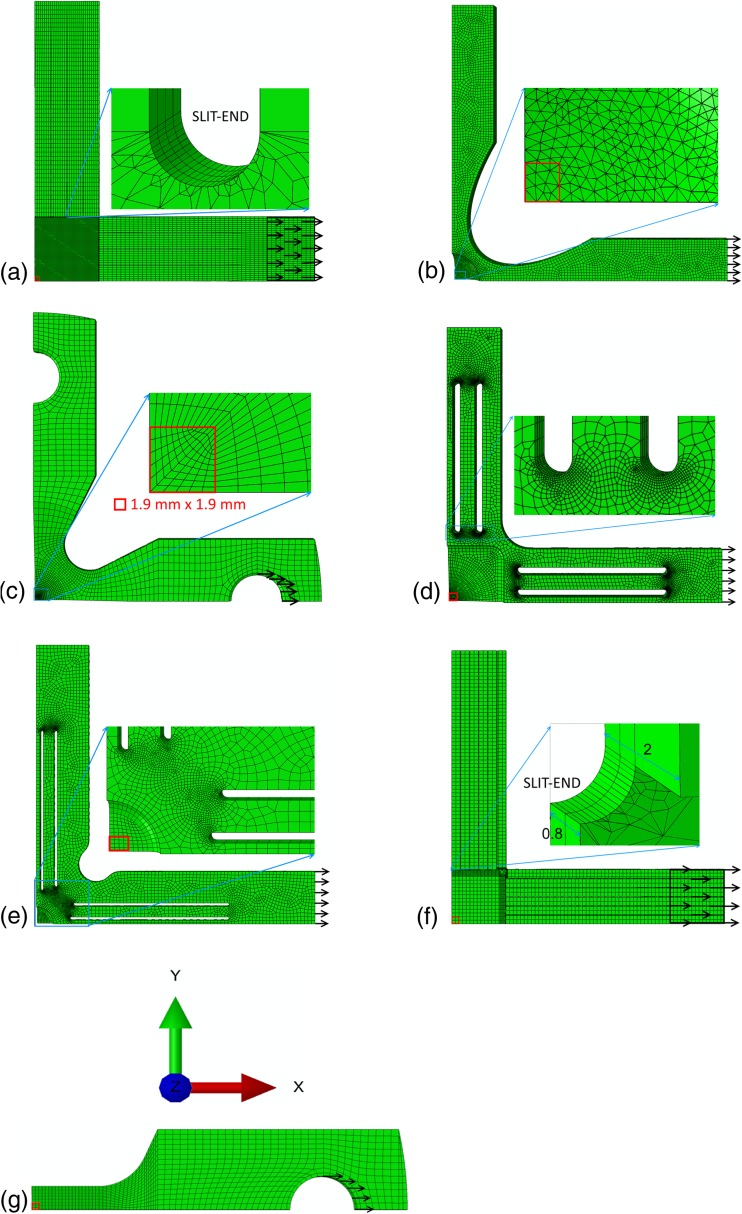
The finite element meshing for the 1/8th (a) SLIT-I, (b) THIN-I, (c) THIN-II, (d) SLIT-THIN-I, (e) SLIT-THIN-II, (f) SLIT-THIN-III, and (g) DB geometries. The red box in each figure indicates the region (1.9 mm × 1.9 mm) used to determine the average strain and stress. The black box in some of the figures represents the surface on which boundary conditions are applied. The black arrows represent the direction of displacement/loading. In some of the figures a zoomed-in picture is provided of a region represented by a blue box. The coordinate system of all the geometries is shown in 3(g)

## Simulation Setup

ABAQUS/Standard software [33] is used to perform the FE simulations for the six cruciform and DB geometries. In order to improve the computational efficiency, only 1/8th of the entire geometry is simulated with symmetric boundary conditions on appropriate surfaces. Figure 3 shows the FE mesh for all the geometries tested in this work. A structured hexahedron mesh is employed with linear 8-node mesh elements (C3D8 in ABAQUS) for the THIN-II and DB geometries as shown in Fig. 3(c), (g). A hexahedron mesh is also used for the SLIT-I and SLIT-THIN-III geometries, except near the slit-ends where a mixed hexahedron/tetragonal mesh is used as shown in the inset of Fig. 3(a), (f). The meshing procedure has been based on the simulations performed by [22] for the SLIT-I geometry and [32] for the SLIT-THIN-III geometry which show that the stress concentrations occur at the slit-ends (inset of Fig. 3(a), (b)). The number of elements for the DB, SLIT-I, THIN-I, THIN-II, SLIT-THIN-I, SLIT-THIN-II, SLIT-THIN-III, and DB geometries are 3333, 29,756, 23,966, 4626, 22,964, 17,408 and 11,336, respectively.

Isotropic elastic properties of 316L stainless steel were used [10]. The plastic response is modeled using the ABAQUS material model that is based on the VM yield criterion and the associated flow rule. The built-in rate-independent combined non-linear isotropic and kinematic hardening law with 5 back-stresses is used. The stress v/s strain curve from the monotonic tensile loading test on DB samples (black curve in Fig. 2) is provided as an input to ABAQUS/Standard. To account for micro-plasticity, the initial yield point is taken at 135 MPa. The ABAQUS/Standard algorithm uses this experimental curve to fit the back-stress parameters; manual parameter fitting is not required. Figure 2 also shows the VM stress v/s strain curve fitted by ABAQUS FE simulation (red line). As can be seen, the fitted and experimental curves have a good match. It should be noted that since the present work does not deal with strain path changes, using an isotropic hardening model instead of a combined hardening model with 5 backstress parameters will not result in significant differences in the predicted stress v/s strain curves. Changing the number of backstress parameters, however, affects the fitting procedure and in this case the combined hardening model with 5 backstress terms provides the best fit.

The experiments on the THIN-II and DB samples are performed under load control, therefore load control is also used to simulate these geometries. The simulated forces are applied as surface tractions on the inside of the holes in the arms of these geometries using a linear ramp. This is illustrated in Figs. 3(c) and (g). The SLIT-I and SLIT-THIN-III geometries are designed to be deformed using clamps attached to the arms. These geometries were deformed under displacement control. Therefore, a linearly ramped displacement on the surface in contact with the clamps is applied in the FE simulations. The remaining geometries have also been deformed under displacement control, as illustrated in Figs. 3(a), (b), (d), (e), and (f).

To facilitate comparison between the different geometries, the strain values for all geometries reported in all the line plots will be averaged on the surface of a 3.8 mm × 3.8 mm area at the center of the gauge area (this becomes 1.9 mm × 1.9 mm area for the 1/8th geometry as shown in the inset of Fig. 3(c) for the THIN-II geometry) and the stresses will be averaged along the thickness of the sample beneath this area. The averaging procedure is motivated from the in-situ neutron diffraction experiments in the work of [10] where surface strains are measured using DIC and the neutrons measurements are obtained from the 3.8 mm × 3.8 mm × 3 mm volume.

## Results

### Experimental Validation of the FE Procedure

**Fig. 4:**
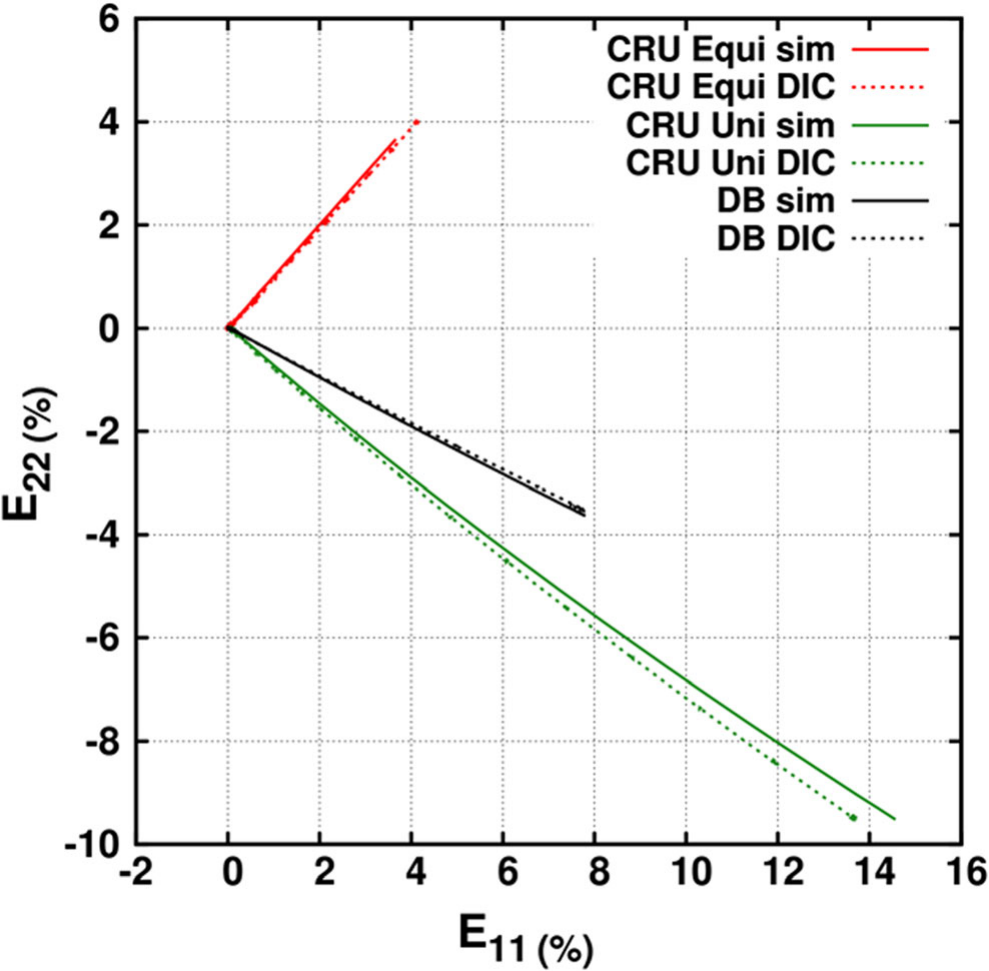
Comparison between simulated (sim) macroscopic strains and the DIC strains for tensile loading on DB samples, and uniaxial (Uni) and equibiaxial (Equi) loading on THIN-II samples

The following set of simulations are carried out for the THIN-II and DB samples and compared with experimental data from [10]: uniaxial tensile loading of DB sample to ~16.4 kN, uniaxial tensile loading along axis 1 of the THIN-II to 50kN and equibiaxial tensile loading of THIN-II (axis 1 and 2) to 50kN on each axis. Figure 4 shows a good match between the two in-plane strain components *E*_11_ and *E*_22_ from the three simulations and the experiments. Minor differences between them may be due to the tolerances in gauge thickness (range of 0.1 mm) associated with manufacturing the samples.

### Plastic Deformation in the Cruciform Geometries

The predicted gauge stress and strain evolution for the six geometries are compared. All the geometries are subjected to uniaxial deformation along direction 1 whereas the arms along direction 2 are kept free i.e. at zero force. The THIN-II cruciform simulations are performed under load control with a linearly ramped force *F*_1_ = 100kN (2000 steps). All the remaining cruciform simulations are performed under displacement control. A linearly ramped displacement is applied at the end of the arm along direction 1. The simulations are stopped when the equivalent plastic strain reaches 40% at the location of the highest stress concentration in each geometry.

**Fig. 5:**
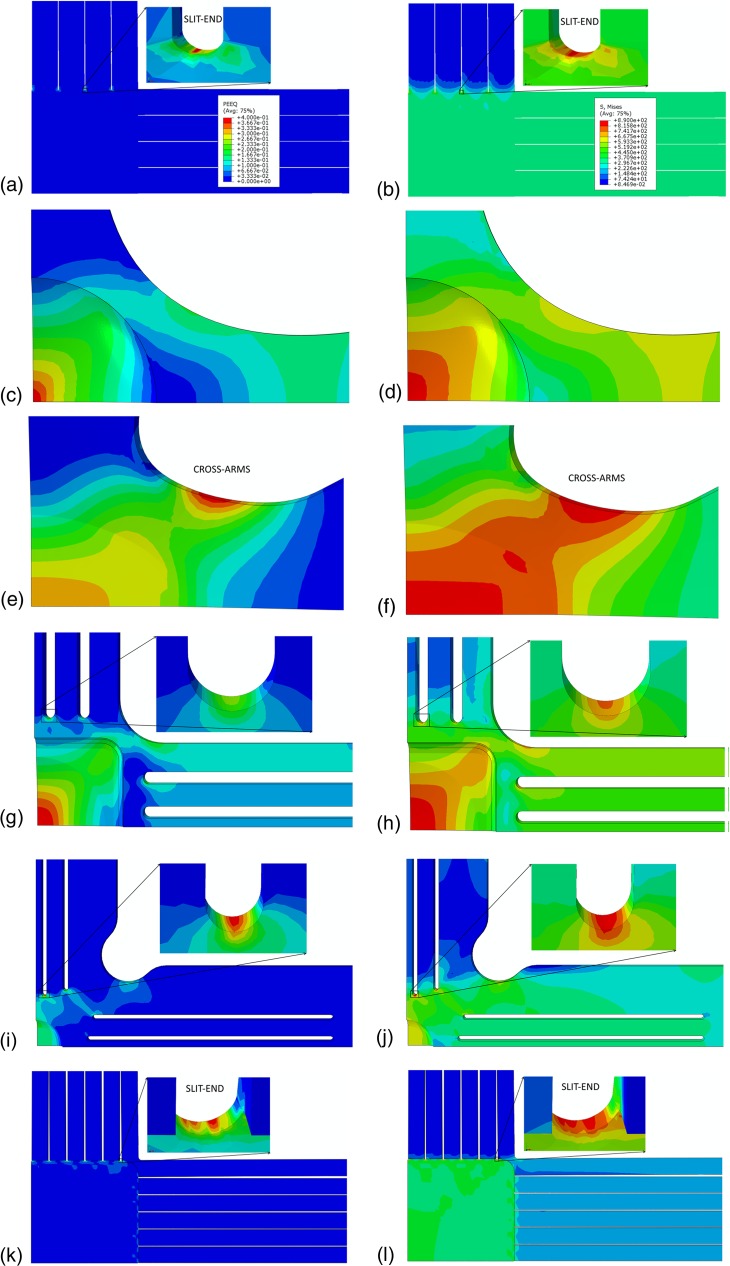
(a, c, e, g, i, k) Equivalent plastic strain and (b, d, f, h, j, l) VM stress distributions in the (a, b) SLIT-I, (c, d) THIN-I, (e, f) THIN-II, (g, h) SLIT-THIN-I, (i, j) SLIT-THIN-II and (k,l) SLIT-THIN-III geometries when the equivalent plastic strain reaches 40% at the location of highest stress concentrations. The in-set in (a, b, k, l) show the zoomed-in slit-ends where the highest stress concentrations occur for the SLIT-I and SLIT-THIN-III geometries. The legends for (c, e, g, i, k) and (d, f, h, j, l) are the same as those shown in (a) and (b), respectively

Figure 5 shows the contour plots for the distribution of equivalent plastic strain and VM stress in and near the gauge region of the six cruciform geometries. The highest stress concentration occurs at the end of the second outermost slit for the SLIT-I, center of the gauge area for the THIN-I, cross-arms for the THIN-II, gauge area for the SLIT-THIN-I, the innermost slit for the SLIT-THIN-II, and the outermost slit for SLIT-THIN-III (shown in Figure 5). For the SLIT-I and SLIT-THIN-III geometries, the VM stress and the equivalent plastic strain are negligible in comparison with those at the location of the slit-ends. For the SLIT-I and SLIT-THIN-III geometries, the location of the slits and their small radius of curvature (0.1 mm) at the slit-ends are responsible for the large difference in the stresses at slit-ends and cruciform gauge area. Furthermore, for the SLIT-THIN-III geometry, the effect of gauge area thinning by a factor of 2.5 to increase the gauge strain is overshadowed by the stress concentrations at the slit-end. For the SLIT-THIN-II geometry, the combined effect of the larger radius of curvature at slit-end, increased distance from the slit-end and gauge area, larger arm-to-gauge thickness ratio of 3.33 and the notch in the arms, results in higher gauge equivalent plastic strain in comparison to the SLIT-I and SLIT-THIN-III geometries.

The THIN-II geometry, has the same arm-to-gauge thickness ratio of 3.33 as the SLIT-THIN-II geometry. However, the VM stress and equivalent plastic strain near the center of the gauge region are comparable to those at the cross arms (Figures 5(e), (f)). This indicates that the gradual thickness reduction from 10 mm in the arms to 3 mm in the gauge region, larger gauge area and the absence of slits play a role in the improved gauge stress. Note that the cross-arms have a variable thickness, which is greater than the gauge area but lesser than the arms. For the THIN-I and SLIT-THIN-I geometries, the maximum equivalent plastic strain and von Mises stresses occur near the center of the specimen. These geometries have an arm-to-gauge thickness ratio of 6.67 and 5.33, respectively. For the THIN-I geometry, the region with the highest stress concentration is smaller in comparison to the SLIT-THIN-I geometry. This is a consequence of the sharp thickness change in the gauge region of the THIN-I geometry.

From these results, we can conclude that a large arm-to-gauge thickness ratio is necessary to obtain the highest stress concentrations in the gauge area, irrespective of the presence of slits or notches at cross-arms. Furthermore, for the slit geometries, the slit width should be comparable to the gauge thickness to avoid high stress concentrations at the slit ends. In addition, the cross-arms should be thicker than the gauge area to reduce the stress concentrations.

### Gauge Strain and Stress Evolution during Uniaxial Deformation in Cruciform and DB Samples

**Fig. 6:**
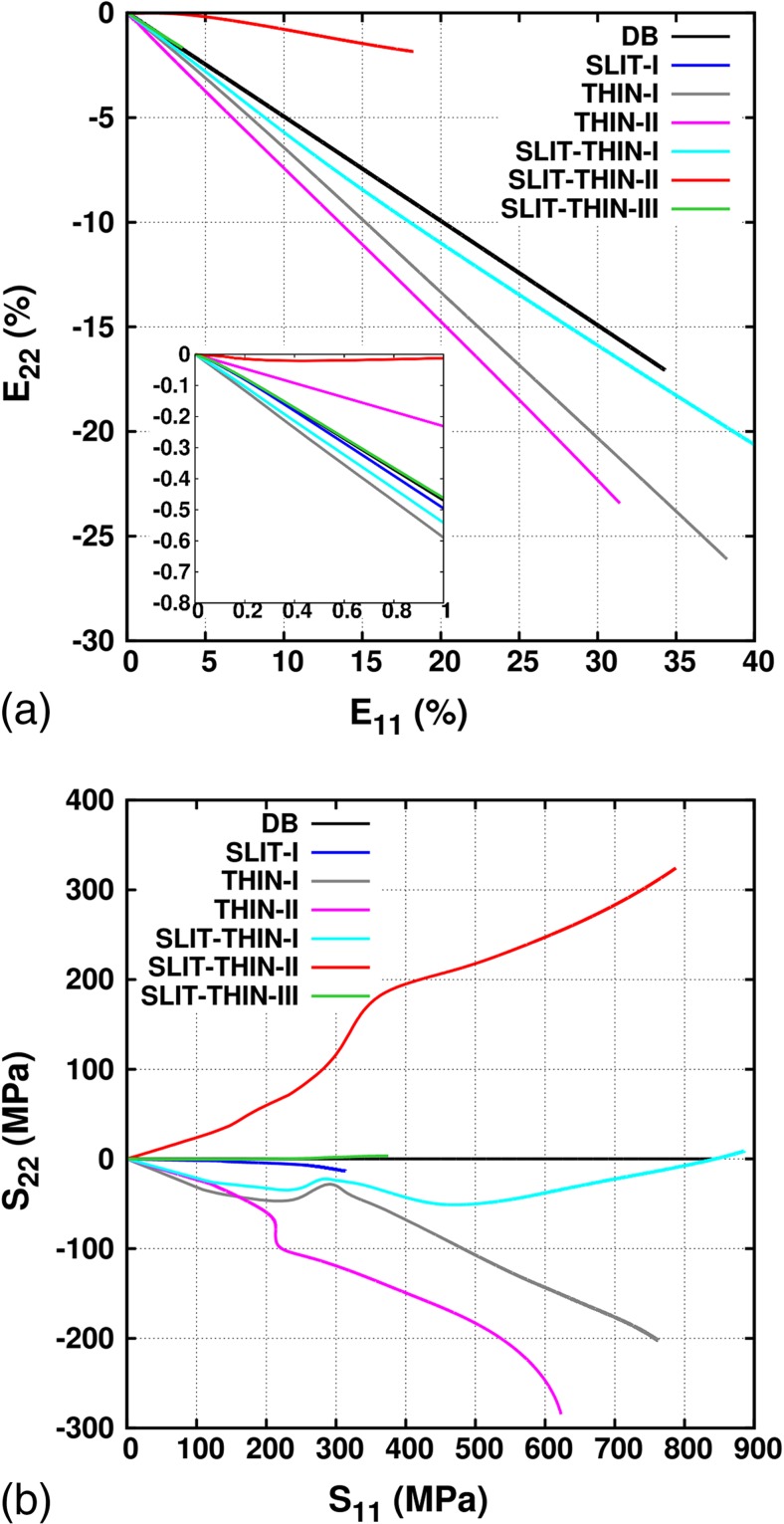
(a) Evolution of macroscopic total strain *E*_22_ as a function of *E*_11_ and (b) Cauchy stress component *S*_22_ as a function of *S*_11_ for the six cruciform geometries and the DB sample (DB)

In this section, the gauge stress and strain evolutions for the six cruciform geometries and the DB are studied during uniaxial loading. The results are shown up to the point where a maximum 40% equivalent plastic strain is reached at the locations of the highest stress concentrations. Figure 6(a) shows the plot of the predicted total gauge strain component *E*_22_ as a function of *E*_11_ for all samples; the DB results are obtained from the simulations performed in section Experimental Validation of the FE Procedure. The ratio $$ \raisebox{1ex}{${E}_{22}$}\!\left/ \!\raisebox{-1ex}{${E}_{11}$}\right. $$ for the DB and cruciform geometries in the elastic (limited to *E*_11_ = 0.1% as shown in inset of Figure 6(a)) and plastic regimes are shown in Table 1. In the elastic regime, for the SLIT-I and SLIT-THIN-III geometries, $$ \raisebox{1ex}{${E}_{22}$}\!\left/ \!\raisebox{-1ex}{${E}_{11}$}\right. $$ is similar to the Poisson’s ratio, however, for the remaining cruciform geometries, it is significantly different. At the end of loading, the SLIT-I, SLIT-THIN-I and SLIT-THIN-III geometries result in a ratio $$ \raisebox{1ex}{${E}_{22}$}\!\left/ \!\raisebox{-1ex}{${E}_{11}$}\right. $$close to −0.5. Whereas, this ratio is less than −0.5 for the THIN-I and THIN-II geometries and more than −0.5 for the SLIT-THIN-II geometry. These results indicate that the SLIT-I, THIN-I, THIN-II and SLIT-THIN-I geometries must have a compressive stress component in the direction 2 normal to the loading direction, whereas SLIT-THIN-II must have a tensile stress component along direction 2. The SLIT-THIN-III geometry should have nearly the same stress state as the DB sample.

**Table 1:** The strain and stress ratios *E*_22_/*E*_11_ and *S*_22_/*S*_11_ in the elastic regime and in the plastic regime at the end of loading for the DB and six cruciform geometries

Ratio geometry	*E*_22_/*E*_11_		*S*_22_/*S*_11_	
	Elastic	Plastic	Elastic	Plastic
DB	-0.31	-0.5	0	0
SLIT-I	-0.33	-0.51	-0.019	-0.045
THIN-I	-0.58	-0.68	-0.35	-0.27
THIN-II	-0.5	-0.67	-0.23	-0.46
SLIT-THIN-I	-0.49	-0.52	-0.21	0.009
SLIT-THIN-II	-0.081	-0.1	0.24	0.41
SLIT-THIN-III	-0.31	-0.48	-0.0043	0.008

Figure 6(b) shows the in-plane stress component *S*_22_ as a function of *S*_11_ for all the geometries. Simulations have revealed that the out-of-plane stress component (*S*_33_) is negligible in comparison with the in-plane stress components. In the elastic regime, the stress ratio $$ (R)\ \raisebox{1ex}{${S}_{22}$}\!\left/ \!\raisebox{-1ex}{${S}_{11}$}\right. $$ is constant for all the geometries and shown in Table 1. Clearly, *S*_22_ is negligibly small for SLIT-I and SLIT-THIN-III but not for the remaining cruciform geometries. Amongst the latter, the SLIT-THIN-II geometry has a tensile stress component along direction 2 while the remaining have a compressive component; the tensile component along direction 2 is counter-intuitive because under uniaxial loading along direction 1, one would expect a compressive component along the transverse direction 2. Immediately following the on-set of plasticity, *R* decreases for the SLIT-I and THIN-II geometries and increases for the remaining geometries. Between *S*_11_ = 200 and 400 MPa, the stress evolution becomes highly non-linear for the THIN-I, THIN-II, SLIT-THIN-I and SLIT-THIN-II geometries. The ratio *R* at the end of loading is also shown in table 1. These results imply that the coupling between the forces in the arms is weak in the SLIT-I and SLIT-THIN-III geometries, intermediate in the SLIT-THIN-I geometry and strong in the THIN-I, THIN-II and SLIT-THIN-II geometries.

The SLIT-I and SLIT-THIN-III geometries are ideal for decoupling the gauge stresses and having negligible transverse stress components under uniaxial loading, however, due to the high stress concentrations at the slit ends these are not suitable to achieve more than a few percent plastic strain in the gauge area. On the other hand, the remaining geometries are suitable to achieve large gauge strains but suffer from a non-linear coupled biaxial gauge stress state at the center of the sample. Furthermore, for the same type of material, the stress evolution in the elastic and plastic regime is significantly different.

### Non-linear Gauge Stresses during General Biaxial Loading

**Fig. 7:**
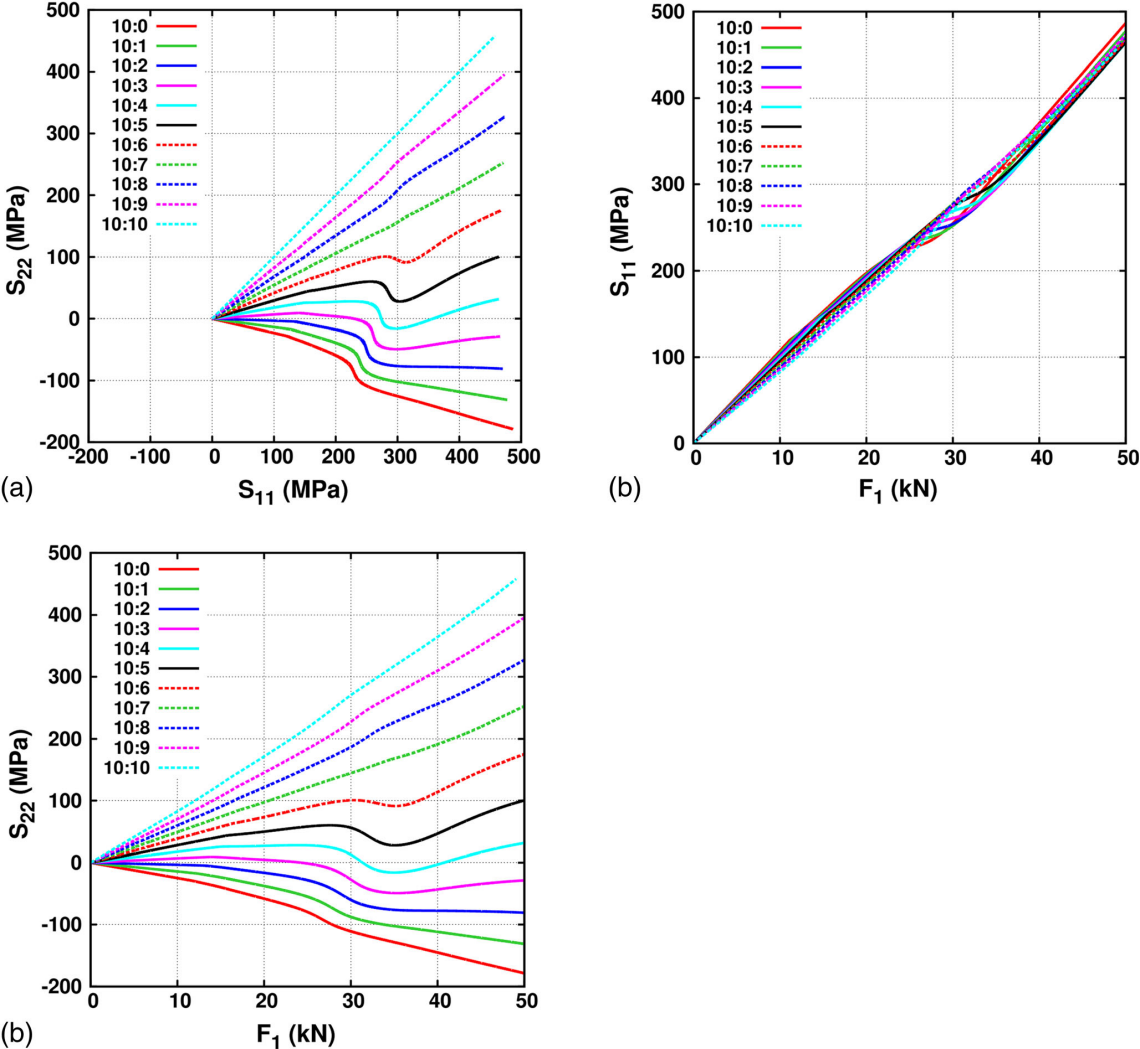
(a) *S*_11_ v/s *S*_22_, (b) *S*_11_ v/s *F*_1_ and (c) *S*_22_ v/s *F*_1_, for *F*_1_:*F*_2_ equal to 10:0, 10:1, 10:2, 10:3, 10:4, 10:5, 10:6, 10:7, 10:8, 10:9 and 10:10

In this section, the non-linearity in the *S*_22_ v/s *S*_11_ evolution is further analyzed for general biaxial loading for the THIN-II geometry. The THIN-II geometry is analyzed because the model predictions have been validated at two extremes of in-plane tensile loading i.e. uniaxial and equibiaxial loading; thus, providing a higher confidence on the accuracy of the predicted results under other biaxial loads. Furthermore, the THIN-II geometry has been used during in-situ neutron diffraction studies in [10, 11]. The non-linear macroscopic stress evolution of the THIN-II geometry influences the inter (type-II) and intra (type III) granular stresses. This is evidenced from the non-linear kink in the lattice strain evolution during uniaxial loading of the THIN-II sample [10, 11]. In the following, the macroscopic stress evolution in the THIN-II sample is studied for 10 different loading ratios *F*_1_:*F*_2_ – 10:0, 10:1, 10:2, 10:3, 10:4, 10:5, 10:6, 10:7, 10:8, 10:9 and 10:10, while *F*_1_ is always increased from 0 to 50kN. The results for the in-plane gauge stress components and the stress-force relationships are shown in Figure 7.

In the elastic regime, the linear stress-force relationship i.e. *S*_11_ = *aF*_1_ − *bF*_2_ and *S*_22_ =  − *bF*_1_ + *aF*_2_ [27–29], is tested. For all loadings, the values for *a* and *b* are found to be constant and equal to 10,750 (*m* ^−2^) and 2500 (*m* ^−2^), respectively. This is in accordance with the work of Hoferlin et al. [27]. The elastic-plastic transition is characterized by a kink in *R* and in the stress-force relationships. The kink evolves differently for different load ratios: it is very pronounced in all three plots for the force ratios 10:0 to 10:6 and less pronounced for the force ratios 10:7 to 10:10. Once full plasticity sets in, the non-linear deviations between the in-plane stresses and stress-force relationships become less pronounced.

Figure 7(b) shows the evolution of *S*_11_ as a function of *F*_1_. Since for all the loading ratios *F*_1_ is ramped to 50kN, the evolution of *S*_11_ is similar for all the load ratios. A closer inspection shows that in *S*_11_ the point of inflection only appears for load ratios between 10:0 to 10:6. In addition, with increasing magnitude of *F*_2_ from a load ratio between 10:0 up to 10:6, the inflection point tends to occur at higher values of *S*_11_. This is because the stress evolution follows the von Mises plastic behavior. Figure 7(c) shows that the evolution of *S*_22_ as a function of *F*_1_ is qualitatively similar to the evolution of *S*_22_ with respect to *S*_11_.

**Fig. 8:**
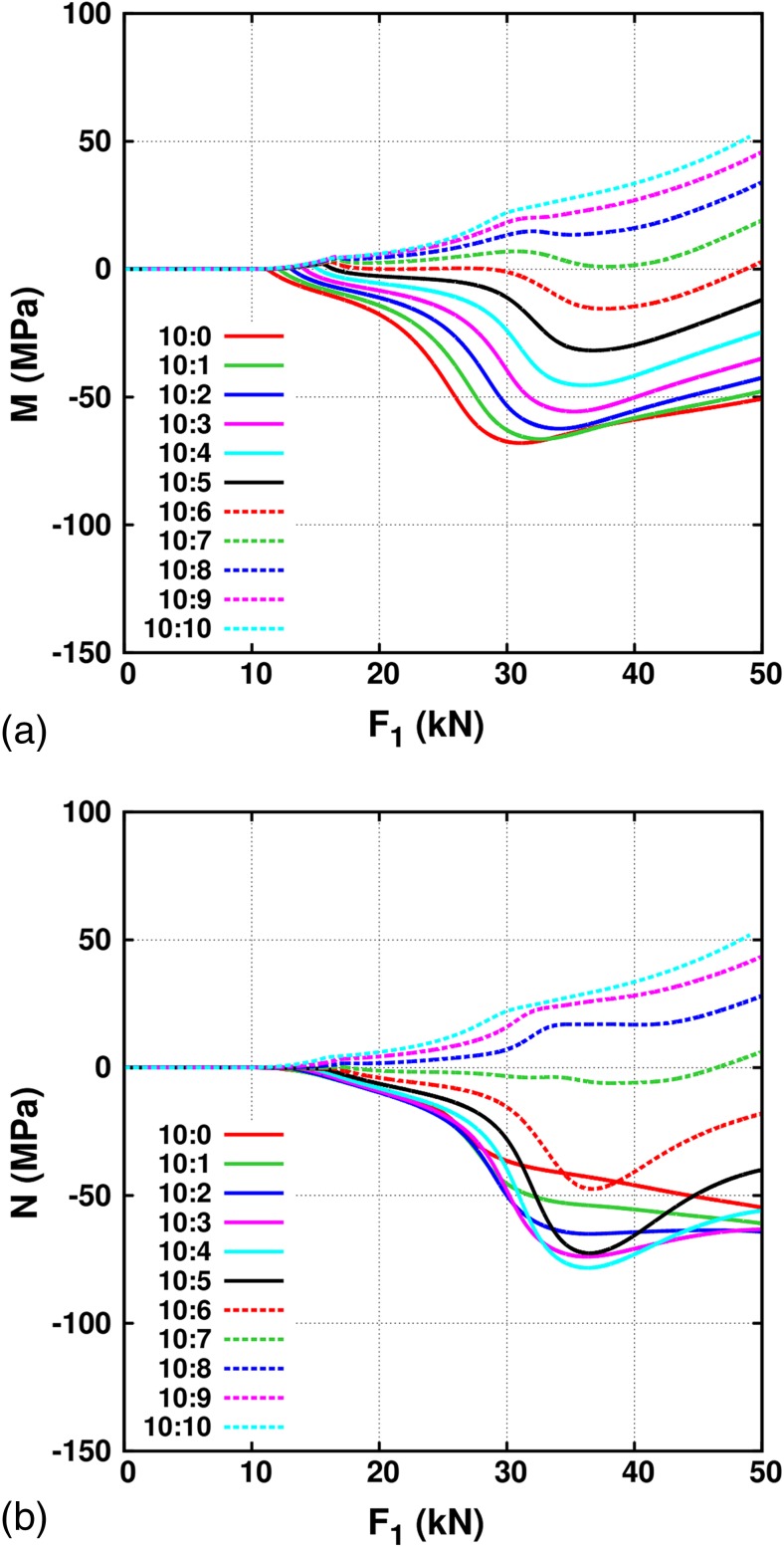
Deviation from the linear behavior in the elastic regime for both the in-plane stress components, (a) $M=\left[{S}_{11}-{F_1}^{\ast }{\left(\frac{S_{11}}{F_1}\right)}_{elastic}\right]$ and (b) $N=\left[{S}_{22}-{F_1}^{\ast }{\left(\frac{S_{22}}{F_1}\right)}_{elastic}\right]$, in the gauge area as a function of the force *F*_1_ in the arm for different load ratios *F*_1_ : *F*_2_ for the THIN-II geometry

In order to better understand the non-linear behavior for different load ratios, Figure 8 shows the deviation from linear evolution of the elastic regime for each stress component i.e. $M=\left[{S}_{11}-{F_1}^{\ast }{\left(\frac{S_{11}}{F_1}\right)}_{elastic}\right]$ and $N=\left[{S}_{22}-{F_1}^{\ast }{\left(\frac{S_{22}}{F_1}\right)}_{elastic}\right]$ as a function of the applied force *F*_1_. The nature of the non-linearity varies significantly as function of the applied force. There are three interesting observations to be made. First, although the kink during the non-linear behavior of *S*_22_ vs *S*_11_ in Figure 7(a) appears to be most pronounced for the load ratio of 10:5, the largest deviation from the linear elastic behavior in *S*_11_ occurs for uniaxial loading i.e. 10:0. The largest deviation from linearity in *S*_22_ occurs first for the load ratio 10:4 at *F*_1_ = 36kN and then for 10:2 at 50kN. Second, equibiaxial loading results in a non-linear evolution of the stress components as a function of the applied force. Third, the load ratio of 10:6 results in a linear evolution of *S*_11_ with the same slope as in the elastic regime up to *F*_1_ = 30kN. This is at the beginning of the elastic-plastic transition. After *F*_1_ = 30kN, *S*_11_ deviates away from the linear behavior. For the load ratio of 10:7, the evolution of *S*_22_ as a function of *F*_1_ is found to be nearly linear till the end of loading. The origin of these counter-intuitive stress evolutions under different load ratios lies in the geometry of the sample. These should vary depending on the cruciform shape and size, and the material properties.

A more general outcome of these simulations is that decoupling the cruciform gauge stresses and computing them as force divided by area is not possible for any stress ratio *R*. Furthermore, this coupling will be different for different geometries. This reinforces the need to use FE simulations to predict the gauge stress state.

### Non-linear Gauge Stress Evolution for Different Hardening Behavior

**Fig. 9:**
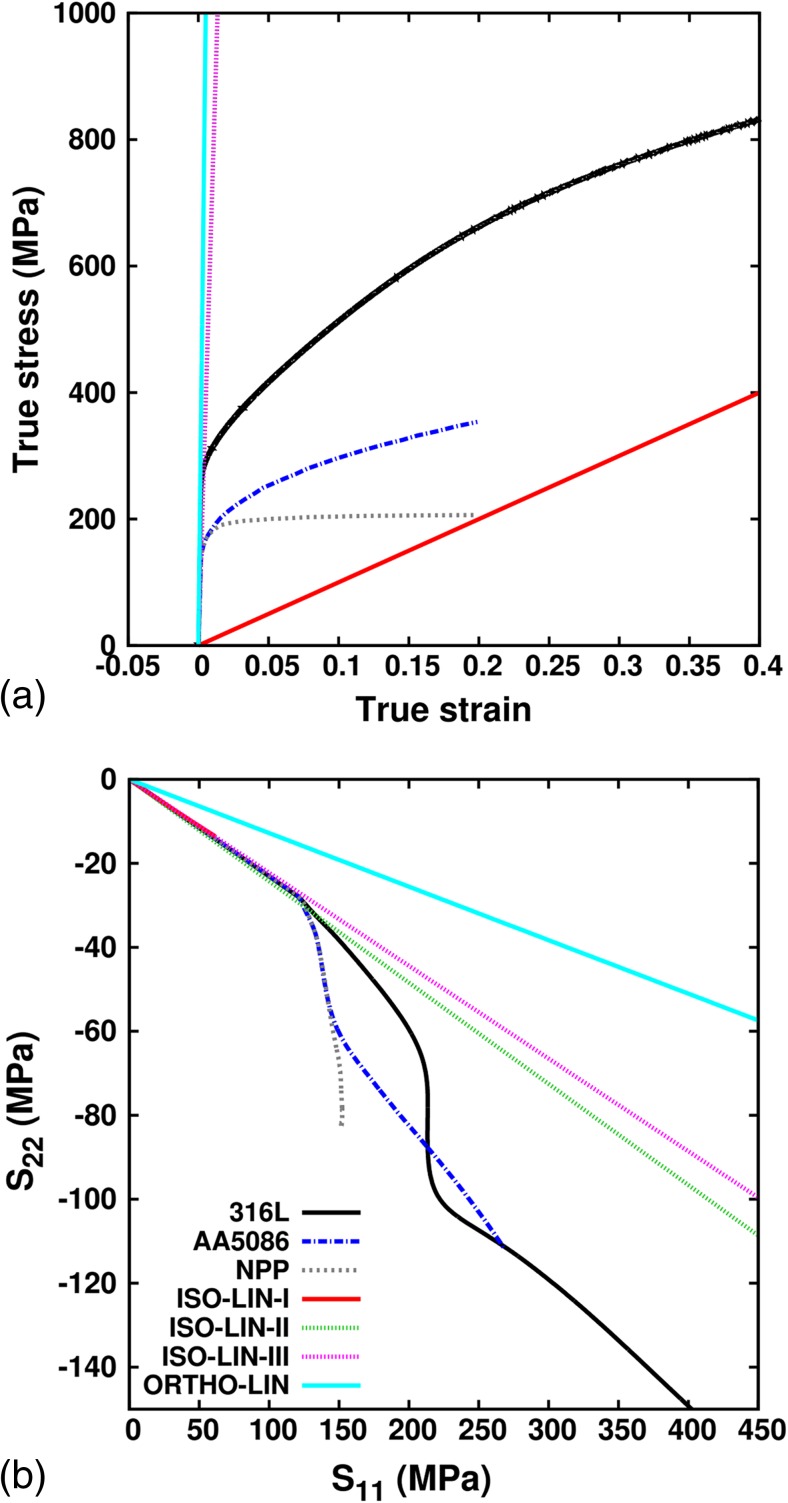
(a) True stress v/s strain curve from FE simulations of DB samples and (b) *S*_22_ v/s *S*_11_ curves for the THIN-II geometry for the (i) 316L, (ii) AA5086, (iii) NPP, (iv) ISO-LIN-I, (v) ISO-LIN-II, (vi) ISO-LIN-III (overlaps ISO-LIN-II) and (vii) ORTHO-LIN materials. The legend for both (a) and (b) is shown in (b)

In this section, we study the gauge stress evolution for the THIN-II cruciform geometry during uniaxial loading for different materials. Figure 9(a) shows the true stress v/s true strain curve for six new materials in addition to the 316 L stainless steel: (i) Aluminum alloy AA5086, similar to the one from [31], with isotropic elastic properties *Y* = 73 GPa but a slightly modified *ν* = 0.31, yield stress at 135 MPa and maximum plastic strain of ~20%, (ii) a nearly perfectly plastic (NPP) pseudo-material with the same elastic properties, yield stress and maximum strain as AA5086, but with a maximum stress of 206 MPa at a maximum plastic strain of ~20%, (iii) an artificial isotropic linear elastic material with *Y*=1GPa and *ν* = 0.31 (ISO-LIN-I), (iv) an artificial isotropic linear elastic material with *Y* = 73 GPa and *ν* = 0.12 (ISO-LIN-II), (v) an artificial isotropic linear elastic material with *Y* = 73 GPa and *ν* = 0.45 (ISO-LIN-III) and (vi) an artificial orthotropic linear elastic material (ORTHO-LIN) with the engineering constants *Y*_1_ = 190GPa, *Y*_2_ = 73 GPa, *Y*_3_ = 190 GPa, *ν*_12_ = 0.31, *ν*_13_ = 0.31, *ν*_23_ = 0.31, *G*_12_ = *G*_13_ = *G*_23_ = 65 GPa. For the ABAQUS simulations of the ORTHO-LIN material, a local coordinate system needs to be defined for each element in the FE mesh. For simplicity, the local coordinate system of all the elements is defined as the global coordinate system. Figure 9(b) shows *S*_22_ as a function of *S*_11_ for the THIN-II geometry for all the materials in this work. Note that the results for the ISO-LIN-I material are only shown until *S*_11_ =  ~ 60 MPa. Beyond this stress, there are significant elastic deflections in the arms at the location of applied force due to the very low stiffness of ISO-LIN-I. This results in a non-linear evolution of the *S*_22_ v/s *S*_11_ which is not representative of the elastic deformations of the THIN-II geometry made from other stiffer materials.

Focusing first on the stresses in the elastic regime, under uniaxial loading we have *S*_11_ = *aF*_1_ and *S*_22_ = *bF*_1_. This gives *R* = *S*_22_/*S*_11_ = *b*/*a*. Next, the Hooke’s law for an orthotropic elastic material with a negligible out-of-plane stress component gives *E*_11_ = *S*_11_/*Y*_1_ − *ν*_12_ *S*_22_/*Y*_2_ and *E*_22_ = *S*_22_/*Y*_2_ − *ν*_21_ *S*_11_/*Y*_1_. Combining the last three equations gives,

1 $$ R=\left(\frac{Y_2}{Y_1}\right)\left(\frac{\raisebox{1ex}{${E}_{22}$}\!\left/ \!\raisebox{-1ex}{${E}_{11}$}\right.+{\nu}_{12}}{1+{\nu}_{21}\raisebox{1ex}{${E}_{22}$}\!\left/ \!\raisebox{-1ex}{${E}_{11}$}\right.}\right) $$

Note here that the −*E*_22_/*E*_11_ is the “effective Poisson’s ratio” for the cruciform geometry and it is not equal to *ν*_12_. This implies that in the elastic regime the biaxial stress ratio *R*, and thus the ratio of the equivalent area measures *b*/*a*, are dependent on the material’s elastic properties. For an isotropic material with *Y* = *Y*_1_ = *Y*_2_ and *ν* = *ν*_12_ = *ν*_21_, equation (1) reduces to $$ R=\left(\frac{\raisebox{1ex}{${E}_{22}$}\!\left/ \!\raisebox{-1ex}{${E}_{11}$}\right.+\nu}{1+\nu\ \raisebox{1ex}{${E}_{22}$}\!\left/ \!\raisebox{-1ex}{${E}_{11}$}\right.}\right) $$. Interestingly, for all the simulated isotropic elastic materials with the same Poisson’s ratio have the same *R* value equal to −0.232. This result suggests that for an isotropic material, *R* depends only on the Poisson’s ratio. This implies that the “effective Poisson’s ratio” i.e. $$ \raisebox{1ex}{${E}_{22}$}\!\left/ \!\raisebox{-1ex}{${E}_{11}$}\right. $$ for a given cruciform geometry should also depend only on the Poisson’s ratio. Therefore, all the isotropic materials with the same Poisson’s ratio should give the same *R*, irrespective of the Young’s modulus. Next, *R* is computed for the ISO-LIN-II and ISO-LIN-III materials having different Poisson’s ratio. These materials, whose Poisson’s ratios are 0.12 and 0.31, respectively, result in an *R* value that are ~5% less and ~5% more, respectively, than the *R* value for the isotropic linear elastic materials with *ν* = 0.31. The ORTHO-LIN material results in *R* =  − 0.129 which results in a 44.4% difference with respect to the isotropic linear elastic materials with *ν* = 0.31. Note that for the ORTHO-LIN material, the difference in Young’s moduli *Y*_1_ and *Y*_2_ also results in a difference in the Poisson’s ratio *ν*_12_ and *ν*_21_; from the relationship *ν*_*ij*_/*Y*_*i*_ = *ν*_*ji*_/*Y*_*j*_, we have *ν*_21_ = 0.12. These results, along with equation (1), imply that *a* and *b*, which have dimensions *m* ^−2^, are dependent on the material properties. This is fundamentally different from the gauge stress-applied force relationship in DB samples, where in the elastic regime this relationship is solely dependent on the DB gauge cross-section.

Following the on-set of plasticity, the evolution of *S*_22_ v/s *S*_11_ varies according to the hardening behavior of the material. An evolution trend is however not obvious. Simulations were also performed for the THIN-I, SLIT-THIN-I and SLIT-THIN-II geometries (results not shown). Similar to the THIN-II geometry, an evolution trend in *S*_22_ v/s *S*_11_ depending on the hardening behavior was not evident.

Engineering metals and alloys, that are macroscopically nearly elastic isotropic, often have a Poisson’s ratio that falls in the range 0.25–0.35. Geometrically same cruciform samples constructed from these materials will have nearly the same stress ratio *R* in the elastic regime. For these materials, *R* will strongly depend on the cruciform geometry and not the elastic properties of the material. A simple uniaxial DB test, along with the strain ratio *E*_22_/*E*_11_ from cruciform samples, is sufficient to obtain the material parameters required for analytically approximating the stress ratio *R*. For anisotropic materials, such as cold rolled Mg-alloys, in the elastic regime *R* will depend on the cruciform geometry shape and the degree of anisotropy; the full macroscopic anisotropic elastic stiffness tensor would be necessary to analytically obtain the stress ratio *R*. In the plastic regime, *R* varies according to both the cruciform geometry and the hardening behavior of the material with no obvious trend, thus making it difficult to analytically obtain its value. This further underlines the need to perform FE simulations to predict their stress evolution.

## Discussion

In the following, we propose cruciform selection criteria to reduce the non-linear gauge stress coupling and to improve the gauge plastic deformation based on the cruciform geometries simulated in the previous section.

1. Symmetric/asymmetric geometry: In the works of [8, 30], the SLIT-THIN-I geometry was originally designed to be a one-sided geometry due to the complexity associated with the design. However, one-sided geometries could suffer from shear and bending stresses in the central region. All the cruciform geometries simulated in this work are symmetric two-sided geometries. The results show negligible shear stresses and out-of-plane normal stress components in comparison to the in-plane normal stresses. The cruciform should be designed symmetric along all three directions.
2. Number of slits and slit width: As seen from the simulations of SLIT-I and SLIT-THIN-III geometries in section 4.2, a relatively large number of slits results in homogenizing the stress field within the gauge area. These results also show that a slit width smaller than the cruciform thickness causes very high stress concentrations at the slit-ends. In contrast, for the SLIT-THIN-I geometry, which has a slit width of 2.2 mm and slit-ends ~3 mm away from the gauge area, the highest stress concentrations occur in the gauge area. However, the biaxial stress ratio is appreciably large and the stress distribution is relatively less homogeneous. Based on these results, the slit-widths should be kept nearly the same size as the gauge thickness and should be as close as possible to the gauge area to obtain a homogeneous stress distribution.
3. Gauge thickness: The gauge thickness should be sufficiently smaller than the arm thickness in order to ensure that the stress concentrations occur in the center of the sample. Amongst the cruciform geometries studied in this work, those with an arm-to-gauge thickness ratio greater than 5 have the highest stress concentration occurring in the gauge area. Note that when the region with reduced thickness encompasses the cross-arms, the ratio of the cross-arm-to-gauge thickness also becomes important.
4. Thickness gradient and gauge area: Cruciform geometries with gradual thickness reduction and larger gauge surface area have shown the highest stress concentrations and more homogeneous stress distribution near the center in comparison to geometries with steep thickness reduction and smaller surface area. The cruciform design should account for this aspect.
5. Cross-arm thickness and shape: Amongst the simulated cruciform geometries, those with cross-arms having the same thickness as the arms and thicker than the gauge area, do not result in stress concentrations in the gauge area.

**Fig. 10:**
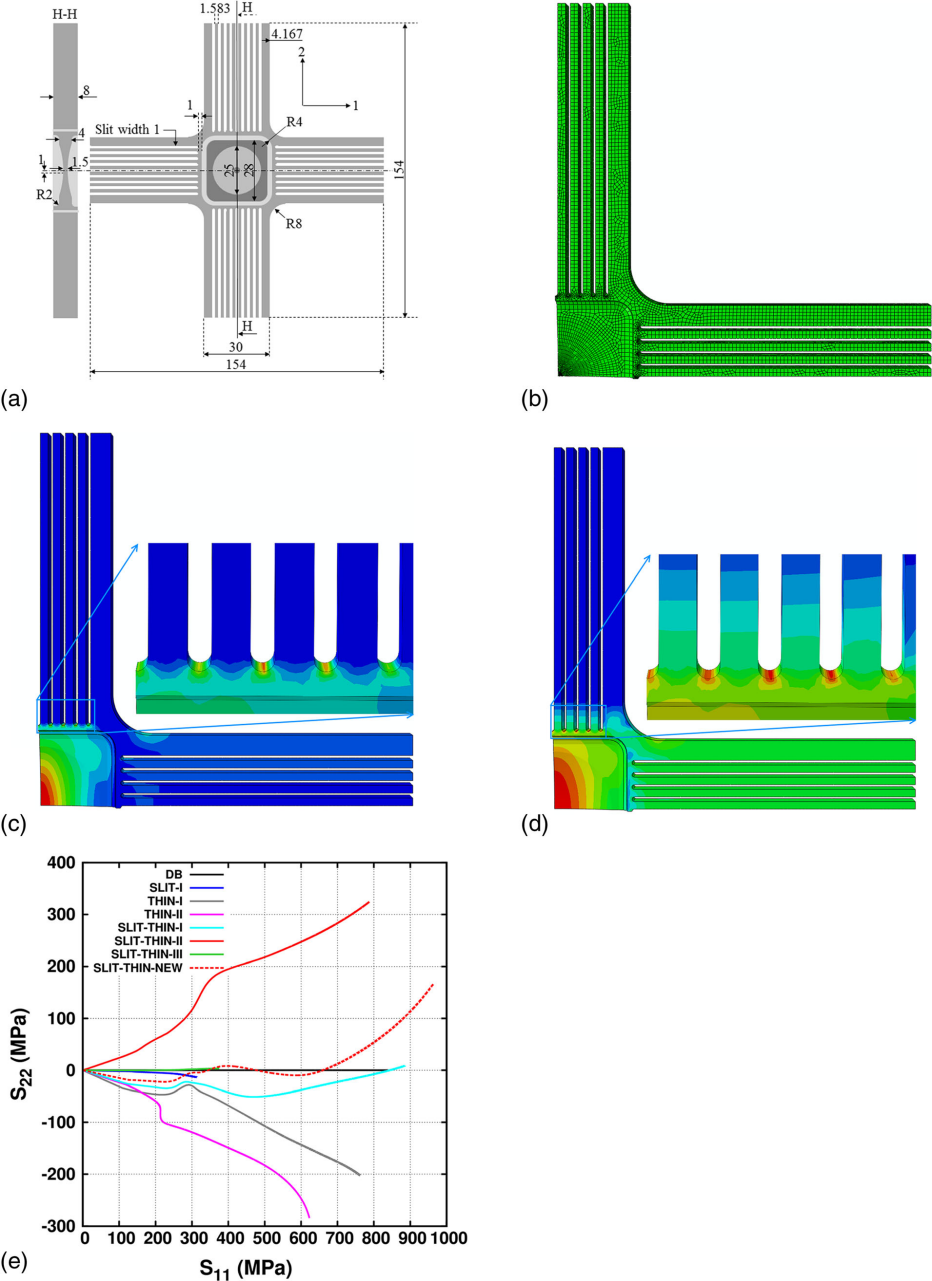
(a) SLIT-THIN-NEW geometry, (b) FE mesh, (c) equivalent plastic strain distribution, (d) von Mises stress distribution, and (e) comparison of *S*_22_ v/s *S*_11_ with other cruciform geometries

Based on these geometry selection criteria, and the cruciform geometries simulated in this work, we propose a cruciform geometry design (non-optimized) for the 316L steel that minimizes the non-linear coupling of gauge stresses while attaining large plastic strains in the center of the gauge region. This is achieved by combining the geometric features from SLIT-THIN-I and SLIT-THIN-III geometries. Figure 10(a) shows the new geometry design, henceforth known as SLIT-THIN-NEW. This geometry has the same arm dimensions, gauge size and area, and cross-arm shape and size as the SLIT-THIN-I geometry. The only difference is the number of slits, slit-width and slit-ends. A total of 9 slits of thickness 1 mm each are added per arm. On one end, all the slits terminate at 1 mm from the region of the first thickness reduction. In the opposite direction, similar to the SLIT-THIN-III geometry, the slits are drawn throughout the length of the arm. This geometry is simulated under uniaxial loading along direction 1. The ABAQUS FE mesh has 20,530, C3D8 mixed hexahedron and tetrahedron elements. The FE mesh is shown in Fig. 10(b). The simulation is stopped when a 40% equivalent plastic strain is first reached anywhere in the geometry. This occurs at the gauge area of the SLIT-THIN-NEW geometry. Figures 10(c) and (d) show a snap-shot of the equivalent plastic strain and von Mises stress distribution in the SLIT-THIN-NEW geometry at the end of the simulation. The simulated *S*_22_ v/s *S*_11_ curve is shown in Fig. 10(e).

Results show that the SLIT-THIN-NEW geometry has the highest equivalent plastic strain in the gauge area of the sample throughout the simulation. The stress concentration is initially highest in the gauge area, however, after *S*_11_ ≈ 700 MPa, it shifts to the third slit-end from the right in the arm along direction 2. In the elastic regime, the SLIT-THIN-III demonstrates a reduced stress ratio *R* in comparison to the SLIT-THIN-I geometry; although not as much as the SLIT-THIN-III geometry. Following the on-set of plasticity, the absolute value of ratio *R* approaches 0. The value of *R* fluctuates around 0 up to *S*_11_ ≈ *S*_*mises*_ = 700 MPa. Following this, the highest stress concentration shifts from the gauge area to the slit-end. In addition, *R* rapidly increases until the end of the simulation. At *S*_11_ ≈ *S*_*mises*_ = 700 MPa, the obtained equivalent plastic strain at the center of the gauge area is ~20%.

Amongst all the cruciform geometries simulated in this work, the SLIT-THIN-NEW geometry is the better geometry to obtain high gauge equivalent plastic strain with the lowest non-linear coupling between the arms but only up to 700 MPa. The SLIT-THIN-NEW geometry can be further optimized by tuning the slits, gauge area and cross-arms. However, the results strongly indicate that designing a single cruciform geometry for all kinds of materials is not feasible when the additional requirements include decoupling the non-linearly evolving in-plane gauge stresses, homogeneous stress distribution, and achieving the highest stresses and large plastic strains in the gauge area.

## Conclusion

In this work, an FE simulation study was undertaken to highlight the role of cruciform shaped sample geometry on the stress evolution within the gauge area. Six cruciform shaped sample geometries are studied: (i) the ISO standard slit geometry – SLIT-I, (ii) the elliptical cross-arm steeply thinned geometry with no slits – THIN-I, (iii) the circular cross-arm gradually thinned geometry with no slits THIN-II, (iv) the two-step gradually thinned geometry with slits – SLIT-THIN-I, (v) the uneven slit, circular notched and sharply thinned geometry – SLIT-THIN-II, and (vi) the modified ISO standard slit geometry – SLIT-THIN-III. The gauge stress and strain evolution for these geometries were compared with DB (dog-bone) samples under uniaxial loading. Then the THIN-II geometry was tested under different biaxial tensile loads for different materials. Based on these studies, cruciform geometry selection criteria were suggested. Using these criteria, a new cruciform geometry (SLIT-THIN-III) was proposed that allowed to achieve medium plastic strain in the gauge area while significantly reducing the non-linear coupling of gauge stresses during uniaxial loading. The main conclusions of this study are as follows:

1. In the elastic regime, the SLIT-I and SLIT-THIN-III geometries result in uniaxial stress state at the center of the gauge region. In contrast, THIN-I, THIN-II, SLIT-THIN-I and SLIT-THIN-II geometries show a linear coupling between the forces in the arms and in-plane gauge stresses in the elastic regime such that *S*_11_ = *aF*_1_ + *bF*_2_ and *S*_22_ = *aF*_2_ + *bF*_1_. A uniaxial tensile load along one of the arms results in a stress component along the in-plane direction normal to the loading direction. This transverse component is tensile for the SLIT-THIN-I geometry and compressive for the THIN-I, THIN-II and SLIT-THIN-II geometries. The transverse stress component can have a significant contribution in defining the gauge stress state. This makes it difficult to analytically compute the gauge stresses as a function of the applied force. The magnitude of the transverse stress component significantly varies according to the cruciform geometry and the macroscopic elastic anisotropy of the material.
2. Following the on-set of plasticity, the THIN-I, THIN-II, SLIT-THIN-I and SLIT-THIN-II geometries result in a non-linear coupling between gauge stresses and applied forces in the arms. Its nature strongly depends on the cruciform geometry, applied biaxial load ratio and elastic-plastic properties of the material. A material, geometry, and applied load ratio dependent trend on the non-linear stress evolution couldn’t be established.
3. In SLIT-I and SLIT-THIN-III, important stress concentrations are developing at the slit ends limiting the plastic strains that can be reached in the gauge region. These geometries are therefore not suitable to study the mechanical behavior of materials with low work hardening rates. In the THIN-II and SLIT-THIN-II geometries, the stress concentrations occur at the cross-arms and slit-ends, respectively. However, these are comparable to the gauge stresses. Consequently, relatively large plastic deformation can be achieved in the gauge area. The THIN-I and SLIT-THIN-I geometries result in the largest von Mises stress and equivalent plastic strains in the gauge area.
4. A new geometry is designed based on the knowledge gained from simulating the six geometries and novel cruciform geometry selection criteria. The new geometry helps reduce the non-linear gauge stress coupling while attaining moderate gauge plastic strains.
